# 368. A Phase 3, Randomized, Double-blind, Parallel-group Trial to Evaluate the Immunogenicity and Safety of AV7909 for Post-exposure Prophylaxis of Anthrax in Healthy Adults

**DOI:** 10.1093/ofid/ofad500.438

**Published:** 2023-11-27

**Authors:** Gideon Akintunde, Bojan Drobic, Lisa Bedell, Lu Gan, Julia Kim, Marinda Beah, Isaac Ghinai, Santiago Barona Collado

**Affiliations:** Emergent Biosolutions, Gaithersburg, Maryland; Emergent Biosolutions, Gaithersburg, Maryland; Emergent Biosolutions, Gaithersburg, Maryland; Emergent Biosolutions, Gaithersburg, Maryland; Emergent Biosolutions, Gaithersburg, Maryland; Emergent Biosolutions, Gaithersburg, Maryland; Emergent Biosolutions, Gaithersburg, Maryland; Emergent Biosolutions, Gaithersburg, Maryland

## Abstract

**Background:**

This was intended as a pivotal safety and immunogenicity Phase 3 trial of AV7909 (Anthrax Vaccine Adsorbed, Adjuvanted) in healthy adults. AV7909 was developed for post-exposure prophylaxis of disease following suspected or confirmed exposure to *Bacillus anthracis.*

**Methods:**

A Phase 3, randomized, double-blind, parallel-group study (NCT03877926) was conducted to evaluate immunogenicity and safety of AV7909 in healthy adults between 18 and 65 years of age. Participants (n=3862) were randomized to receive AV7909 (intramuscularly at 0, 2 weeks) or BioThrax^®^ (subcutaneously at 0, 2, 4 weeks), a comparator vaccine. Immunogenicity of AV7909 was assessed using pre-specified 50% neutralizing factor (NF_50_) protective thresholds at Day 64 (seven weeks after the last dose of AV7909, five weeks after last dose of BioThrax) generated by a toxin neutralizing antibody (TNA) assay, and by evaluating non-inferiority of AV7909 to BioThrax. Safety was evaluated by collection of solicited reactogenicity for seven days after each vaccination and reports of adverse events (AEs) for up to one year after last vaccination.

**Results:**

The first primary immunogenicity endpoint was met, wherein the lower bound (LB) of two-sided 95% confidence interval (CI) for the proportion of participants (64.4%) with TNA NF_50_ ≥0.56 was above the pre-defined criterion of ≥40%. The primary non-inferiority immunogenicity endpoint was met, wherein the LB of two-sided 95% CI of difference in the proportion of participants with TNA NF_50_≥0.29 in AV7909 group compared to BioThrax group was above the pre-defined criterion of -15% (i.e., 20.5%). The incidence of AEs, including serious AEs, AEs leading to study discontinuation, and confirmed AEs of special interests (i.e., autoimmune in origin) were similar between the AV7909 and BioThrax groups. AEs related to injection site were the most common for both vaccines. Most participants reported solicited reactogenicities and AEs that were either Grade 1 or Grade 2 severity.

**Conclusion:**

The study met the prospectively defined success criteria for primary endpoints, demonstrating a protective level of immunogenicity and non-inferiority of AV7909 to BioThrax vaccine. AV7909 administered IM two weeks apart was well tolerated in healthy adults.

**Disclosures:**

**Gideon Akintunde, Director Clinical Development**, Emergent Biosolutions: Employee **Bojan Drobic, Director Clinical Development**, Emergent Biosolutions: Employee **Lisa Bedell, Director Biostatistic**, Emergent Biosolutions: Employee **Lu Gan, Director Biostatistic**, Emergent Biosolutions: Employee **Julia Kim, Scientist Clinical Development**, Emergent Biosolutions: Employee **Marinda Beah, Manager Clinical Trials Development**, Emergent Biosolutions: Employee **Isaac Ghinai, Medical Director**, Emergent Biosolutions: Employee **Santiago Barona Collado, Medical Director**, Emergent Biosolutions: Employee

